# Proceedings of the 12^th^ International Meeting on Neuroacanthocytosis, Cohen Syndrome, and Other VPS13-Related Disorders

**DOI:** 10.5334/tohm.1124

**Published:** 2026-01-09

**Authors:** Fabrizio Vacca, Binnaz Yalcin, Lars Kaestner, Adrian Danek, Kevin Peikert, Ruth H. Walker, Muhammad Ansar

**Affiliations:** 1 Department of Ophthalmology, University of Lausanne, Jules-Gonin Eye Hospital, Fondation Asile des Aveugles, Lausanne, Switzerland; 2 Institut NeuroMyoGene-PGNM UCBL-CNRS UMR5261 INSERM U1315, Lyon, France; 3 Université Bourgogne-Europe, INSERM U1231, 21000, Dijon, France; 4 Theoretical Medicine and Biosciences, Medical Faculty, Saarland University, 66421 Homburg, Germany; 5 Dynamics of Fluids, Experimental Physics, Saarland University, 66123 Saarbrücken, Germany; 6 Neurologische Klinik und Poliklinik, LMU Klinikum, LMU München, Munich, Germany; 7 Translational Neurodegeneration Section “Albrecht Kossel”, Department of Neurology, Rostock University Medical Center, University of Rostock, Germany; 8 Center for Transdisciplinary Neurosciences Rostock (CTNR), University Medical Center Rostock, Rostock, Germany; 9 United Neuroscience Campus Lund-Rostock (UNC), Rostock site, Germany; 10 Department of Neurology, James J. Peters Veterans Affairs Medical Center, Bronx, NY, USA; 11 Department of Neurology, Icahn School of Medicine at Mount Sinai, New York City, NY, USA; 12 Advanced Molecular Genetics and Genomics Disease Research and Treatment Centre, Dow University of Health Sciences, Karachi, Pakistan

**Keywords:** Neuroacanthocytosis, bridge-like, lipid transfer protenins, Cohen syndrome, XK-disease, McLeod syndrome

## Abstract

The 12^th^ International Meeting on Neuroacanthocytosis, Cohen Syndrome, and other VPS13-related Disorders was held on September 12^th^–14^th^, 2025, at the Jules Gonin Eye Hospital in Lausanne, Switzerland. This long-standing series of international symposia has traditionally focused on neuroacanthocytosis syndromes and associated disorders. The program further broadened its scope to include Cohen syndrome, reflecting the growing recognition of shared molecular features and common unsolved questions across VPS13-related disorders.

The aim of the meeting was to present the latest updates in the field, from both clinical and basic science perspectives, and to facilitate collaboration and exchange of ideas among researchers, clinicians, and the patient community. An important aspect of these meetings is the active involvement of patients, their relatives and caregivers, who were invited to attend scientific sessions, in addition to participating in parallel patient-oriented sessions. A total of 20 oral communications were presented in eight scientific sessions accompanied by two keynote lectures, short talks by selected poster presenters, and the 2025 “Glenn Irvine Prize” award lecture.

## Introduction

VPS13A disease (formerly chorea-acanthocytosis; due to bi-allelic variants in *VPS13A*) and XK disease (formerly McLeod syndrome; due to variants of *XK*) are rare neurodegenerative diseases, classified together as the “neuroacanthocytosis syndromes” [1]. Recent advances have identified the VPS13 proteins (VPS13A-D) as members of the novel family of bridge-like lipid transfer proteins (BLTPs), which facilitate lipid transport between subcellular organelles at membrane contact sites [2,3], thus grouping the associated diseases under a common pathomechanistic umbrella [1]. In light of these developments, the focus of the 2025 conference was expanded to include perspectives of cellular functions of this novel protein family, and to include clinicians and basic science researchers focusing upon VPS13B-D and the related BLPT1, in addition to VPS13A and XK diseases, with the aims of sharing insights into potential common disease mechanisms and hence potential therapeutic mechanisms.

VPS13A and XK diseases are remarkably similar in their clinical phenotype, which primarily reflects involvement of the basal ganglia, especially of the caudate nucleus. The resulting presentation is similar to that of Huntington’s disease, with the additional features of peripheral neuropathy and myopathy, hepatomegaly, and seizures seen in approximately half of patients. Patients with XK disease have the additional involvement of cardiac muscle, with cardiac disease often being a presenting feature and a cause of morbidity and mortality, and with a specific erythrocyte antigen presentation (absent Kx and reduced Kell antigens) which has implications for blood transfusion. Elevation of creatine kinase and transaminases is diagnostically informative, and indeed is more so than the initially eponymous acanthocytosis. The diverse aspects of these two disorders have led to diverse areas of research, including; clinical biomarkers to facilitate diagnosis; structural and biochemical human postmortem studies; the generation of mouse models to examine the motor phenotype, studies of red cell membrane properties; and cell biology studies in yeast and other cell culture models.

Bi-allelic variants in the related gene *VPS13B* cause Cohen syndrome, a complex neurodevelopmental disorder characterized mainly by intellectual disability, hypotonia and progressive retinal dystrophy. Variants of the *VPS13C* gene result in a genetic form of Parkinson’s disease, while those of *VPS13D* lead to an ataxic syndrome. Variants of the more recently characterized BLTP1 underlie Alkuraya-Kučinskas syndrome, a severe neurodevelopmental disorder also discussed here.

The conference primarily focused on functions and dysfunctions of proteins from the VPS13 family, in addition to XK, which is closely linked to VPS13A, acting as an associated lipid scramblase. Highlights included recent advances in the structural analysis of BLTP proteins and new insights into the mechanisms of lipid transfer. Additional sessions addressed progress in the development of disease model systems, the identification of novel biomarkers, genetic studies, and pre-clinical therapeutic strategies.

Twenty oral communications were presented in eight scientific sessions, along with 10 talks selected from poster presentations. Keynote lectures were given by Adrian Danek and Pietro de Camilli. The 2025 “Glenn Irvine Prize” lecture was given by Marianna Leonzino, in recognition of her pivotal contributions uncovering the lipid-transfer function of VPS13 proteins. Previous prize recipients are Jae-Sook Park (2021) and Kevin Peikert (2023). This award is given in memory of Glenn Irvine, who, with his wife Ginger, founded the Advocacy for Neuroacanthocytosis Patients in 2002.

The patient-oriented program included presentations were organized by the Advocacy for Neuroacanthocytosis Patients, UK; NA Advocacy, USA; Cohen Syndrome Research foundation, and include presentations by occupational and speech therapists; mental health support; educational sessions on orthopedic and other aspects of Cohen syndrome; personal Reiki sessions; and a theatre performance by an actor living with VPS13A disease, entitled “If I get to Korea, I’ll tell you” (performance in Portuguese, with supporting English written translation).

The symposium was funded by the Swiss National Science Foundation (grants IZSEZ0_237449, IZSTZ0_216615 and 320030_212959), Jules-Gonin Eye Hospital, Fondation Asile des aveugles, Lausanne; Advocacy for Neuroacanthocytosis Patients, UK; NA Advocacy, USA; Cohen Syndrome Research Foundation.

## Keynote Lectures

**Disorders of bulk lipid transfer, an emerging disease category**

Adrian Danek

*Neurologische Klinik und Poliklinik, LMU Klinikum, LMU München, Munich, Germany*

The availability of molecular genetic testing has critically influenced the approach to diagnosing rare and ultrarare conditions such as VPS13A disease and XK disease (previously known as chorea-acanthocytosis and McLeod syndrome, respectively) as well as Cohen syndrome (due to *VPS13B* mutations). The historical umbrella label of “neuroacanthocytosis” no longer is appropriate and must be split up as it is not a final diagnosis and its continued use prevents precise diagnosis for the individual patient. Further, it fails to lump conditions with a shared pathomechanism [1].

Progress in biochemistry and cell biology has led to recognition of bulk transport of lipids via hose-like proteins such as VPS13A and VPS13C as a novel concept. The nomenclature committee of HUGO, the Human Genome Organisation [2], subsequently defined the group of ten “BLTP” genes that code for “bridge-like lipid transfer proteins” with shared structure and the assumption of shared function, thus promising shared treatment approaches.

To be useful for the lumping of disease entities [3], a concept of BLTP-associated conditions must take into account that XK disease clinically presents like a slowly progressing variant of VPS13A disease. The similarity of the two conditions is explained by the fact that the two proteins affected, XK and VPS13A, interact in the bulk transfer of lipids. Other than XK there is a considerable number of BLTP interactors, e.g. FAM177A1 that interacts with VPS13B.

While the lumping of diseases related to BLTP and BLTP interactors is plausible from the science perspective, it poses a problem that practical medicine appears unable to solve. Clinical variability, presentation modes, signs and symptoms, the different ages of onset and modes of genetic transmission preclude simple translation of the concept into practice.

In conclusion, the “BLTP/BLTP interactor diseases” concept challenges basic science to develop a global function parameter for easy diagnosis of these disorders, comparable to biomarkers such as CRP and NFT for e.g. inflammatory and neurodegenerative conditions.

**VPS13 family proteins in physiology and disease**

Pietro De Camilli

*Department of Neuroscience, Department of Cell biology, Yale University School of Medicine, New Haven, Connecticut 06510, USA*

Cellular life requires the transport of membrane lipids between intracellular membranes. This transport is achieved either by vesicular trafficking or by lipid transport proteins (LTPs), which shield lipids within hydrophobic cavities as they travel through the aqueous environment of the cytosol. Until recently, known LTPs were shown to carry only one or a few lipids in their cavity and to move them between membranes by a shuttle-like mechanism. Over the last few years, studies of VPS13 have pioneered the identification of a new class of LTPs that transport lipids through a bridge-like mechanism [1,2].

My talk focused on what we have learned about VPS13A and VPS13C and summarized information on the structure of these proteins generated from cryo-EM studies by the Reinisch lab, also in collaboration with our lab and the Vanni lab. A major site of action of VPS13A is at contacts between the ER and the plasma membrane, where its C-terminal PH domain binds XK. This interaction, however, appears to be highly regulated, as it is not observed in all cells [3]. The major site of action of VPS13C is at contacts between the ER and lysosomes. This interaction is also highly regulated, as it is triggered by lysosomal damage. This finding led to a model according to which VPS13C resides in the cytosol in an autoinhibited state, which is released when its ATG2-C domain binds the damaged lysosomal membrane through an interaction that synergizes (in a coincidence-detection mechanism) with its binding to Rab7 [4,5]. Cryo-EM studies of the VPS13A–XK complex and of VPS13C support the occurrence of inactive (lipid transport–non-permissive) and active (lipid transport–permissive) states of VPS13 proteins which differ because of a major change in the arrayment of the VAB domain relative to the rod-like bridge domain of these proteins. These studies also revealed that VPS13A and VPS13C are constitutively bound to calmodulin, suggesting an important role for Ca²^+^ signaling in their function. Finally, we found that while absence of either VPS13A or VPS13C is compatible with life in mammals, the combined knockout of VPS13A and VPS13C in mice leads to embryonic lethality at mid-gestation. Arrest of embryonic development is due impairment of fetal erythropoiesis, revealing a non redundant role of these two proteins in differentiating erythroblasts [6].

## Session presentations

### Clinical perspectives of VPS13A/XK/Cohen disease

**Clinical Management in VPS13A/XK**

Ruth H. Walker

*Department of Neurology, Icahn School of Medicine at Mount Sinai, New York, NY, USA*

*James J. Peters Veterans Affairs Medical Center, Bronx, NY, USA*

The neuroacanthocytosis syndromes, VPS13A and XK diseases, are characterized by neurological, cognitive, and psychiatric symptoms attributable to progressive degeneration of the basal ganglia, in addition to other features including seizures, peripheral neuropathy and myopathy. In XK disease there are often additional cardiac issues, and hematological aspects related to the rare, McLeod blood type.

Management is focused upon identifying the symptoms which need to be addressed, and optimizing psychological and physical functioning with individually tailored therapies. Speech and swallowing, and nutrition are often affected early, especially in VPS13A disease, and require particular specialist attention.

Movement disorders, seizures, and psychiatric issues should be managed with conventional pharmaceutical therapies, and often need to be individualized. Patients should be evaluated on an ongoing basis, at least 1–2 times per year, to assess the efficacy and need for therapies, and to determine whether any new symptoms, e.g. parkinsonism, could be medication side effects or attributable to disease progression.

Deep brain stimulation (DBS) may be useful in reducing hyperkinetic movements, especially those affecting the lower face and tongue in VPS13A disease. The benefits seem to wane after a few years, maybe due to disease progression or target migration, although more sustained effects have been reported.

An inter- and multi-disciplinary team approach, including long-term caregiver support, is essential for these complex, and multifaceted disorders. While the vast majority of clinical providers will never have previously seen patients with these specific conditions, experience with other movement disorders such as Huntington’s and Parkinson’s disease, can provide guidance.

**Update on VPS13A Disease**

Kevin Peikert^1-3,6^, Dajana Großmann^1^, Jan Suchy^1^, Sipan Mahmo^1^, Zacharias Kohl^4^, Julian Conrad^5^, Uwe Walter^6,7^, Lucia de Franceschi^8^, Adrian Danek^9^, Ruth H. Walker^10,11^, Lars Kaestner^12,13^, Andreas Hermann^1-3,7^

*^1^Translational Neurodegeneration Section “Albrecht Kossel”, Department of Neurology, Rostock University Medical Center, University of Rostock, Germany*

*^2^Center for Transdisciplinary Neurosciences Rostock (CTNR), University Medical Center Rostock, Rostock, Germany*

*^3^United Neuroscience Campus Lund-Rostock (UNC), Rostock site, Germany*

*^4^Department of Neurology, University of Regensburg, Regensburg, Germany*

*^5^Division for Neurodegenerative Diseases, Department of Neurology, Universitaetsmedizin Mannheim, University. of Heidelberg, Mannheim, Germany*

*^6^Department of Neurology, Rostock University Medical Center, University of Rostock, Germany*

*^7^German Center for Neurodegenerative Diseases (DZNE) Rostock/Greifswald, Rostock, Germany*

*^8^Department of Engineering for Innovative Medicine- DIMI, University of Verona, Verona, Italy*

*^9^Neurologische Klinik und Poliklinik, LMU Klinikum, LMU Munich, Munich, Germany*

*^10^Department of Neurology, James J. Peters Veterans Affairs Medical Center, Bronx, NY, United States*

*^11^Department of Neurology, Icahn School of Medicine at Mount Sinai, New York City, NY, United States*

*^12^Theoretical Medicine and Biosciences, Medical Faculty, Saarland University, Homburg, Germany*

*^13^Dynamics of Fluids, Experimental Physics, Faculty of Natural Science and Technology, Saarland University, Saarbrücken, Germany*

Remarkable scientific advances in our field in recent years have significantly improved our pathophysiological understanding of VPS13A disease. However, there are still major translational roadblocks on the way to the establishment of disease modifying treatments. Limited knowledge on the natural history and the lack of properly validated biomarkers are still obvious barriers for “clinical trial readiness”.

This presentation provides an update on our collaborative efforts in respect to establishment of novel biomarkers, disease phenotyping and case identification. Biomarker candidates include neurofilament in serum, certain phosphatidylethanolamine species in red blood cells, various biophysical properties of red blood cells, and the erythrocyte sedimentation rate. Brain parenchyma sonography might help to distinguish VPS13A and XK disease from other chorea syndromes. Differences in the echogenicity of the basal ganglia could indicate distinct patterns of neurodegeneration and tissue remodeling in VPS13A/XK disease compared to other chorea syndromes. Furthermore, we present first quantitative measurements of XK and VPS13A protein levels in a healthy control cohort, in the related disorders and other neurodegenerative diseases. The VPS13A/XK Western blot is especially helpful for case identification (e.g., when access to genetic testing is limited) and for evaluating variants of unknown significance in the *VPS13A* and *XK* genes.

The talk furthermore emphasizes the urgent need for international cohort building and biobanking.

**The Clinical Perspective of Neuroacanthocytosis**

Hans H. Jung

*Department of Neurology, University Hospital Zürich, Zürich, Switzerland*

Choreoacanthocytosis (ChAc) and McLeod syndrome (MLS), formerly called “core neuroacanthocytosis syndromes”, are exceedingly rare, but also very likely to be underdiagnosed. Acanthocytosis is an unspecific reaction form of RBC and is not obligatorily present in routine blood smear examinations. These neurodegenerative disorders are associated with VPS13A dysfunction in ChAc, and XK dysfunction in MLS. Notably, XK acts as lipid scramblase for VPS13A, suggesting common pathogenetic pathways and therapeutic targets.

Although rare, the diagnosis of VPS13A and XK disease are straightforward. The typical clinical phenotype includes a progressive chorea syndrome similar to Huntington disease, with some particular features such as reduced or absent tendon reflexes and elevated serum CK levels. Confirmation of the diagnosis includes immunohematological evidence of absent Kx RBC antigen and reduced Kell RBC antigens in XK-disease, as well as molecular genetic confirmation of disease-causing mutations in the XK or VPS13A gene. Increasing the awareness in the neurological community by participation and implementation of symposia in national and international congresses is warranted, and the established VPS13 forum is an extremely valuable tool with the potential for a wider audience.

Novel data of eight patients show that the neuropathology of XK disease is characterized by neuronal loss, gliosis and atrophy of the basal ganglia with a severity gradient from the caudate nucleus to the putamen and pallidum, in decreasing order. Intraneuronal vacuoles in the basal ganglia are a microstructural hallmark of advanced disease, suggesting a possible role of lipid pathology. Comprehensive investigation of the largest pathological cohort of XK disease to date allowed for the proposal of a standardized grading system for the neuropathological assessment of XK disease.

Urgently needed is a deeper insight in the pathomechanisms of VPS13A and XK disease to facilitate attempts of drug repurposing. However, gene silencing is not feasible in presumed loss of function mutations. Since double blind randomized studies are not feasible in these ultrarare diseases, future therapeutic studies have to be performed in individual patients or small patient cohorts, where clinical efficacy will be difficult to be determined. Hence, biomarker signals might be crucial to determine promising candidate approaches, necessitating a better understanding biomarker profiles via a central biobank furnished with as much samples as possible (blood, CSF, tissue samples, imaging data).

**Building Bridges to the Future: Insights from Two Decades of Comprehensive Medical Care for Over 140 Cohen Syndrome Patients**

Heng Wang

*DDC Clinic – Center for Special Needs Children, Middlefield, Ohio, USA*

The Amish communities of North America represent unique populations due to their closed communities and small founding populations. There is increased incidence of rare recessive disorders in Amish populations, caused by founder effects. Biological and social aspects of the Amish communities make them ideal participants for studies in population genetics and genomic medicine. Beginning in the 1960s, inherited disorders among Old Order Amish communities were the subject of exceptional observational studies and many conditions still remain subjects of extensive research.

During the last two decades, we have provided comprehensive medical care for 145 Cohen syndrome patients (103 Amish and 42 non-Amish) through DDC Clinic, Center for Special Needs Children in Middlefield, Ohio, one of the largest Old Order Amish settlements in North America. We have adopted the Medical Home concept recommended by the American Academy of Pediatrics making our services accessible, family-centered, continuous, comprehensive, coordinated, compassionate and culturally effective to the patients and affected families, particularly for the local Amish community. The care model we have delivered integrates multiple sub-specialties into primary care which has significantly improved patients’ outcomes. In addition, through the Cohen Syndrome Association, we connect with over 100 additional non-Amish individuals with Cohen syndrome.

We have learned a great deal of knowledge from patients and families through the comprehensive care we provide. The expertise that we have developed in Cohen syndrome from both clinical care and translational research in the last two decades has significantly benefited patients, families and the medical community. We extend our professional services by providing educational resources for both health professionals and the public through scientific publications, GeneReviews, NORD, Orphanet and periodic support group meetings etc. We apply what we have learned back to patient management. This endeavor exemplifies how translational genomic medicine can benefit affected patients and families both within the Old Order Amish communities and the broader international rare disease community.

Working with this large cohort, we are in a unique position as we move forward. Our ongoing work on 1) the natural history and biomarkers study 2) pathological mechanism and intervention of neutropenia and 3) assessments of growth hormone in the improvement of lean body mass and central obesity might shed more light on the understanding and treatment of this disease.

### Bridge-like lipid transfer proteins: Basic science

**The ends of the tunnel: studies of the N- and C-terminal ends of Vps13**

Jae-Sook Park and Aaron M. Neiman

*Department of Biochemistry and Cell Biology, Stony Brook University*

Bridge-like transfer proteins (BLTPs) are defined by a long, hydrophobic channel that spans the cytoplasmic gap between different organellar membranes at contact sites and mediates movement of lipids between the membranes. While this model for BLTP function is now well established, how the activity of BLTPs is regulated remains to be determined. We are investigating the interaction of the C-terminal and N-terminal ends of the Vps13 channel with other proteins to better understand how lipid transport is controlled.

In sporulating yeast cells, Vps13 mediates the transfer of lipids from the endoplasmic reticulum (ER) to the prospore membrane, allowing expansion of the latter compartment and the formation of spores. At this membrane contact, the N-terminal end of Vps13 is associated with the ER and the C-terminus with the prospore membrane, defining a direction of lipid movement from N- to C- terminal through the channel. At the C-terminal end, Vps13 associates with at least three prospore membrane-localized proteins. An Alphafold Multimer prediction of the structure of the complex indicates extensive interactions with partner proteins holding the exit from the Vps13 channel in proximity to the membrane.

By contrast, at the N-terminal end, selective association with the ER can be directly mediated by two short helices within Vps13. Mutagenesis of these helices, as well as replacement of the first RBG repeat with that from other BLTPs, suggest a model in which the N-terminal end of the lipid transfer channel ‘opens and closes’ to allow for lipid entry as well as ER binding and release.

**The Alkuraya-Kučinskas Syndrome – Lipid transport is necessary for neocortical lamination**

Alexandre Reymond

*Center for Integrative Genomics, University of Lausanne, Lausanne, Switzerland*

We previously described the Alkuraya-Kučinskas syndrome, a disorder associated with biallelic variants in *BLTP1* (bridge-like lipid transfer protein; *KIAA1109*). A large fraction of the forty-five Alkuraya-Kučinskas syndrome individuals previously described and of the ten patients identified in the present report, presented with perinatal death or were elected for premature termination of pregnancies (65%, 36 out of 55 cases). Specifically, all carriers of bi-allelic truncating variants died perinatally (21/23; 91%) made exception of a mother and her daughter, who carry a homozygous noncanonical splice donor variant suggested to lead to less severe consequences via leaky splicing. Carriers compound heterozygote for a truncation and a missense variant and carriers of biallelic missense variants in *BLTP1* exhibited lower rates of perinatal death (5/11 (45%) and 10/21 (48%), respectively). Probands presented with hydrocephalus (76%; 34 out of 45 cases), cerebellar anomalies (63%; 26/41), corpus callosum agenesis (59%; 26/44), ventriculomegaly and arthrogryposis. Homozygous ablation of mouse *Bltp1* resulted in similar preweaning lethality.

Because of its structural similarity with VPS13A-D (vacuolar protein sorting 13 homolog A-D), the 5093 residue-long BLTP1 protein (NM_001384125.1) was suggested to form a hydrophobic tunnel that bridges the endoplasmic reticulum to the plasma membrane. To model the possible effect of the identified missense variants, we used the experimentally solved cryogenic electron microscopy structure of the first ∼ 1,700 residues of LPD-3, the BLTP1 C. elegans ortholog, in addition to the human AlphaFold model. Their structure consists of 17 repeats of β-groove (RBG), typically composed of five anti-parallel strands and one helix connected by a long loop. Eight of the twenty-three missense variants were identified in individuals that died perinatally suggesting that they are loss-of-function variant. Seven of these variants, are positioned within beta-strands that form the RBG repeats barrel.

To model this syndrome, we engineered Emx1-Cre-mediated conditional knockouts (cKO) in which *Bltp1* expression is only removed in cortical and hippocampal neurons. This restricted ablation of *Bltp1* recapitulated the preweaning lethality observed in the constitutional knockouts, suggesting that lack of *BLTP1* expression in neurons is sufficient to cause death. Homozygous cKO presented a complete agenesis of the corpus callosum, a smaller anterior commissure, a malformed hippocampus and a reduced thickness of the cortical plate with a lack of defined structural layers and absence of Pax6-, Tbr2- and Tbr1-expressing radial glial and intermediate neural progenitors and mature neurons, respectively.

**Architecture of a native bridge-like lipid transport protein complex**

Sarah Clark

*Department of Biochemistry and Biophysics, Oregon State University, Corvallis, OR, USA*

Cells and cellular organelles are surrounded by membranes that are constantly undergoing lipid modification due to processes like cell growth, organelle biogenesis, exocytosis, and phagocytosis. Bridge-like lipid transport proteins (BLTPs) have emerged as key players in all of these processes due to their role in lipid transport. BLTPs localize to membrane contact sites, where they fold into hydrophobic tunnels that are proposed to function like “lipid superhighways” that mediate the bulk transfer of lipids from donor to acceptor membranes. Despite the fundamental importance of BLTPs for cellular function, the mechanism of lipid transfer remains enigmatic. Here, we present the subunit composition and cryo-electron microscopy structure of the native LPD-3 BLTP complex isolated from transgenic C. elegans. Our results suggest a model for how the LPD-3 complex mediates bulk lipid transport and provide a foundation for mechanistic studies of BLTPs.

**The molecular mechanism of lipid transport by bridge-like lipid transfer proteins**

Stefano Vanni

*Department of Biology, University of Fribourg, Switzerland*

*NCCR Bioinspired Materials, University of Fribourg, Fribourg, Switzerland*

Recent breakthroughs in both protein structure determination and artificial-intelligence based predictions have allowed to investigate large macromolecular protein assemblies with unprecedented accuracy down to the atomistic scale. Yet, characterizing and understanding the mechanism of such machineries at the molecular level, and especially in the presence of cellular membranes, remains extremely challenging, owing to the highly dynamic nature of lipid assemblies.

To overcome this limitation, computational methods, such as molecular dynamics (MD) simulations, have emerged as a powerful tool for studying the dynamics and mechanisms of complex processes taking place at cellular membranes. To this end, we have recently developed new computational assays based on molecular simulations and combined them with various experimental approaches (in situ cryo-ET, fluorescence microscopy, in vitro and in vivo functional assays) to investigate how molecular interactions between proteins and lipids modulate cellular processes.

In this contribution, I will show how these developments have allowed us to gain unprecedented molecular insights into lipid transport at membrane contact sites. Specifically, by investigating in a high-throughput fashion the interactions between proteins and lipids, we are starting to unveil the molecular mechanism of lipid transport by bridge lipid transport proteins (BLTPs) of the Atg2-Vps13 family and by BLTP-interacting proteins.

**Genetic and physical interactions of Vps13 lipid transporter with Rsp5 ubiquitin ligase**

J. Kaminska^1^, W. Rzepnikowska^2^, S. Ginikachukwu Ngwube^1^, A. Kochanski^2^, T. Zoladek^1^

*^1^Institute of Biochemistry and Biophysics Polish Academy of Sciences, Warsaw, Poland*

*^2^Neuromuscular Unit, Mossakowski Medical Research Institute Polish Academy of Sciences, Warsaw, Poland*

Human *VPS13A-D* genes, mutations in which cause rare neurodegenerative diseases, are evolutionarily conserved. Yeast *S. cerevisiae* is a good model to study the function of Vps13 proteins because it only has a single *VPS13* gene. Vps13 proteins are responsible for bulk lipid transport at various membrane contact sites. Their localization depends on interactions with specific proteins and lipids, characteristic for different compartments. To find proteins that determine the localization and regulate Vps13 three different approaches were applied: (1) purification of Vps13-TAP from yeast cells and identification of interacting proteins by mass spectrometry; (2) a search for interacting proteins by means of the yeast two-hybrid system; (3) screening for second site suppressors of the sodium dodecyl sulphate (SDS) sensitivity of the *vps13-I2749R* mutant (the *vps13-I2749R* mutation corresponds to the *VPS13A-I2771R* mutation identified in a patient) [1]. Among proteins co-purified with Vps13-TAP was Rsp5, an ubiquitin ligase from the NEDD4 family. The interaction between Vps13 and Rsp5 was confirmed by co-immunoprecipitation. Further analysis showed that Vps13 undergoes ubiquitination, but independently of Rsp5. Furthermore, the lysine 1894 residue, a potential site of ubiquitination, is important for proper functioning of Vps13. The two-hybrid screen identified peptidylpropyl cis-trans isomerases (Cpr6 and Fpr1) as potential candidates for binding to Vps13. The interaction between Vps13 and Fpr1 was confirmed by co-immunoprecipitation. Fpr1 is a functional partner of the calcium-dependent phosphatase calcineurin and of TORC1 complex. Indeed, our previous works have shown that the absence of functional Vps13 causes changes in calcium signaling [2,3]. Moreover, the third approach identified mutations in the *KOG1* gene, encoding a subunit of the TORC1 complex, as suppressor mutations. Experiments to uncover the mechanism of this suppression revealed that a mutant lacking the *VPS13* gene (*vps13Δ*) is resistant to rapamycin and sensitive to caffeine (drugs that affect TORC1), and that the *vps13Δ kog1* mutant has reduced levels of Sch9 kinase, one of the main substrates of TORC1 kinase. In summary, these results indicate that Vps13 proteins may undergo isomerization or various post-translational modifications (ubiquitination and phosphorylation), which may affect the regulation or localization of Vps13. Furthermore, the results suggest that Vps13 absence is detected by various signaling pathways.

### Advances in VPS13A research

**An Update on ‘Red Blood Cells as a Diagnostic Biomarker for Neuroacanthocytosis Syndromes’**

Alexis Darras^1^, Min Qiao^2^, Kevin Peikert^3^, Anne Hecksteden^4^, Thomas John^2^, Emeric Stauffer^5^, Ingrid Muniansi^5^, Benoit Champigneulle^6^, Aurelien Pichon^7^, Michael Furian^8^, Ivan Hancco^9^, Julien V. Brugniaux^6^, Alzbeta Mühlbäck^10^, Elie Nader^5^, Philippe Joly^5^, Tim Meyer^2^, Samuel Verges^6^, Andreas Hermann^3^, Adrian Danek^11^, Philippe Connes^5^, Christian Wagner^2^, Lars Kaestner^2^

*^1^University of Bristol, United Kingdom*

*^2^Saarland University, Germany*

*^3^University Hospital Rostock, Germany*

*^4^University of Innsbruck, Austria*

*^5^University of Lyon, France*

*^6^University of Grenoble, France*

*^7^University of Poitiers, France*

*^8^University Hospital Zürich, Switzerland*

*^9^University of San Martin de Porres, Peru*

*^10^Isar Amper Hospital, Germany*

*^11^Ludwig Maximilian University Munich, Germany*

The erythrocyte sedimentation rate (ESR) is one of the most common and widely used laboratory diagnostic parameters in connection with inflammatory reactions and it is highly likely that everybody has already experienced a determination of this blood parameter [1,2]. A rapid ESR is a non-specific parameter that provides information about the inflammatory process [3,4,5]. Although the origins of this methodology date back to antiquity, the description of the process as the collapse of a percolating gel formed from erythrocytes has only recently been achieved [6,7,8,9]. It was previously proposed but is not yet known whether slow ESR has any medically relevant significance. Here we show a variety of clinical pictures that exhibit a systematically slow ESR (e.g., sickle cell disease, neuroacanthocytosis syndromes, chronic mountain sickness). Using a combination of measured data and physical modelling, we show how the accuracy and significance of ESR data can be increased (SupraESR). Furthermore, we show that the SupraESR and associated parameters can be used for the differential diagnosis of neuroacanthocytosis syndromes. With the slowed ESR, we have a completely new diagnostic parameter based on an established measurement method that is easy to establish and extremely cost-effective. If the ESR is measured automatically, as is often the case today, we have the tool for a low-cost screen for neuroacanthocytosis syndromes that until now was difficult to be determined.

**VPS13 expression in red blood cells**

Christian J. Stevens-Hernandez and Lesley J. Bruce

*Bristol Institute for Transfusion Sciences, NHS Blood and Transplant, Bristol, UK*

The red blood cell (RBC) membrane consists of a lipid bilayer containing numerous integral and peripheral proteins that interact to form complexes. The band 3/ankyrin complex and the protein 4.1/junctional complex attach the membrane to the underlying spectrin cytoskeleton, which forms a hexagonal lattice. VPS13A associates with XK protein (which is linked to Kell protein), β-adducin and β-actin of the junctional complex. There are about 40,000 junctional complexes per RBC but that only 1:2 – 1:10 of these contain Kell, XK and VPS13A. Mutations in VPS13A and XK are associated with chorea-acanthocytosis (ChAc) and Mcleod (MLS) syndromes respectively. ChAc RBCs do not contain VPS13A, and MLS RBCs contain no XK or VPS13A. Both ChAc and MLS RBCs form acanthocytes. VPS13A is a bulk lipid transporter moving lipids between the endoplasmic reticulum and other membranes. As mature RBCs do not contain internal membranes, we wondered whether VPS13A has a role during erythropoiesis. In normal RBCs, we found that VPS13A is highly expressed in early erythropoiesis and levels decline as the cells mature. Whereas XK had low expression in early erythropoiesis that increased with maturation. In ChAc and MLS mature RBCs our data suggests that reticulocyte maturation was normal despite the presence of acanthocytes. Looking back at past proteomic studies we discovered that both VPS13C and VPS13A are expressed in reticulocytes and speculated that VPS13C may compensate for loss of VPS13A during erythropoiesis. ChAc RBCs have been shown to have heightened tyrosine phosphorylation which destabilizes the membrane. Similarly, protein kinase C activity destabilizes the junctional complex but only under certain conditions, such as sickle cell anaemia, *Plasmodium falciparum* infections, RBC vesiculation and in early reticulocytes. In each of these cases the RBC membrane is disrupted, and we speculate that VPS13A may play a role in repair of the RBC plasma membrane in these conditions.

**The effects of membrane contact sites disruption on lipid transfer and calcium homeostasis in iPSC-derived neurons with loss of VPS13A from patients with VPS13A disease**

Dajana Großmann^1^, Emily Fischer^1^, My Uyen Dang Thi^1^, Kevin Peikert^1,2,3^, Andreas Hermann^1,2-4^

*^1^Translational Neurodegeneration Section “Albrecht Kossel”, Department of Neurology, Rostock University Medical Center, University of Rostock, Germany*

*^2^Center for Transdisciplinary Neurosciences Rostock (CTNR), University Medical Center Rostock, Rostock, Germany*

*^3^United Neuroscience Campus Lund-Rostock (UNC), Rostock site, Germany*

*^4^Deutsches Zentrum für Neurodegenerative Erkrankungen (DZNE) Rostock/Greifswald, Rostock, Germany*

Background: Membrane contact sites (MCS) are critically involved in the function and maintenance of different organelles like mitochondria, the endoplasmic reticulum (ER) or peroxisomes. MCS are major hubs for the exchange of calcium ions or lipids. VPS13A is a membrane-bound protein bridging opposing membranes at MCS to enable bulk lipid transfer between organelles. Loss of the VPS13A protein resulting from mutations in the *VPS13A* gene cause VPS13A disease/chorea-acanthocytosis.

Objective: Although MCSs are becoming increasingly important in neurodegenerative disease research, their contribution to neuronal death is still largely unclear. We attempt to investigate the structure and function of MCSs to better understand the consequences of their dysfunction in the context of VPS13A disease.

Methods: Induced pluripotent stem cell (iPSC)-derived neurons from patients harbouring mutations in *VPS13A* were used. Different MCS were quantified by super resolution microscopy using an AxioObserver.Z1 LSM 900 microscope with Airyscan 2 module and a 63x 1.4 NA plan apochromat objective (Zeiss) in live and fixed neurons. We used immunostaining to analyse different organelles. Furthermore, lipid and calcium homeostasis was investigated using specific dyes for live cell imaging.

Results: We observed alterations of lipid distribution and impaired cellular calcium homeostasis of VPS13A deficient neurons. As a consequence, the function of organelles like mitochondria, peroxisomes and lysosomes was affected. Further, we observed alterations of mtDNA, likely resulting from impaired mitochondrial calcium homeostasis, eventually causing activation of cellular stress response pathways. VPS13A deficient neurons displayed increased peroxidation of lipids, likely contributing to the demise of neurons.

Conclusion: The changes in lipid distribution, caused by loss of VPS13A may result in a whole range of problems for many cellular processes. Future research must take into account the entire cell when investigating disruptions to mass lipid transfer at MCS in order to understand how neurodegeneration occurs.

Funding: A. H. is supported by the Hermann und Lilly Schilling-Stiftung für medizinische Forschung im Stifterverband. D.G. received funds from the German Research Foundation (DFG: GR 6326/2-1). DFG Großgeräteantrag für LSM 900 Airyscan microscope (FKZ: INST 264/175-1 FUGG).

### Advances in Cohen syndrome (VPS13B)

**Understanding the genetics of Cohen Syndrome and potential therapeutic options**

Muhammad Ansar

*Department of Ophthalmology, University of Lausanne, Jules-Gonin Eye Hospital, Fondation Asile des Aveugles, Lausanne, Switzerland*

Cohen syndrome (CS) is a rare autosomal recessive neurodevelopmental disorder caused by biallelic variants in VPS13B, with an estimated prevalence of ~50,000 affected individuals worldwide. The clinical spectrum includes progressive retinal degeneration, macular edema, neutropenia, high myopia, cataract, postnatal microcephaly, hypotonia, developmental delay, and intellectual disability. To date, more than 1,200 pathogenic or likely pathogenic VPS13B variants have been reported, highlighting extensive allelic heterogeneity.

Analysis of population genomic constraint metrics reveals that VPS13B tolerates loss-of-function (LoF) variants (pLI = 0; LOEUF = 0.71), similar to VPS13A and VPS13C, whereas VPS13D is LoF-intolerant (pLI = 1; LOEUF = 0.43) and essential for cell viability. Causative missense variants in VPS13B are rare and generally not clustered within specific domains, although some have been reported in the N-terminal Chorein domain. In contrast, several pathogenic missense variants in VPS13D map to exons 66–68, which encode the C-terminal adaptor region encompassing the VAB (β-propeller) domain and the 5′ edge of the adjacent DH–PH cassette. These observations suggest that domain-specific missense changes in both VPS13B (N-terminal Chorein) and VPS13D (C-terminal adaptor) should be interpreted carefully, as they may impair protein function.

To dissect the molecular basis of CS and assess therapeutic avenues, we generated VPS13B knockout (KO) HeLa cells and characterized Vps13b KO mice. Transcriptomic profiling of retinal pigment epithelium of Vps13b KO mice revealed profound dysregulation of pathways, including upregulation of RNA decapping processes and downregulation of antiviral innate immune responses. Given the large size of VPS13B (~12 kb cDNA), which exceeds the packaging capacity of adeno-associated viral (AAV) vectors, we explored alternative gene-based strategies. Antisense oligonucleotide (AON)–mediated exon skipping and truncated constructs targeting repeat β-grasp (RBG) domains were tested for their ability to restore Golgi morphology, a key phenotypic readout of VPS13B function, in KO cells. Of 24 exons screened, two showed promising results, demonstrating that exon removal can yield functional rescue and providing a potential therapeutic option for patients carrying mutations in these exons.

Complementary to these preclinical studies, compassionate-use taurine supplementation in siblings with SLC6A6 variants (encoding the taurine transporter) demonstrated reversal of cardiomyopathy and arrest of retinal degeneration. This finding underscores the therapeutic potential of restoring disrupted metabolic pathways, even later in disease progression, and supports analogous approaches for Cohen syndrome.

**Drug discovery for the treatment of Cohen Syndrome**

Fabrizio Vacca

*Department of Ophthalmology, University of Lausanne, Jules-Gonin Eye Hospital, Fondation Asile des Aveugles, Lausanne, Switzerland*

Cohen Syndrome (CS) is a rare neurodevelopmental disorder caused by mutations in VPS13B gene and affecting approximately 50,000 individuals worldwide. It is characterized by intellectual disability, postnatal microcephaly, progressive retinal dystrophy, and other systemic symptoms. VPS13B belongs to the Bridge-like lipid transport proteins (BLTP) family and is localized in the Golgi complex. The prominent cell-autonomous phenotype observed in CS is the fragmentation of this organelle, which is also assumed to be highly relevant in disease pathogenesis. We developed a robust cell-based assay for high-content compound screening, monitoring the recovery of Golgi morphology in VPS13B KO Hela cells. We used the rescue of this cell-autonomous phenotype as the basis for a microscopy-based high-throughput screening assay. By screening the Prestwick chemical library, we identified several small molecules capable of restoring Golgi morphology. Most of these compounds shared a common mechanism of action, relying on lipid accumulation in acidic organelles due to their cationic amphiphilic properties (CADs).

Lipidomic profiling revealed a reduction in C18-N-acyl sphingolipids as a characteristic feature of VPS13B knockout (KO) cells, a defect that was reversed by the majority of the identified compounds. We tested two compounds, azelastine and raloxifene, in cortical organoids (COs) derived from VPS13B KO human pluripotent stem cells. These organoids exhibited smaller size and reduced neurite outgrowth, reminiscent of the secondary microcephaly observed in CS patients. Treatment with either compound significantly recovered the neurite outgrowth phenotype, reinforcing physiological relevance of the compound effect. We expanded our drug discovery strategy by screening an additional 12,000 compounds including a repurposing library (RPL) of 4,160 compounds and chemical diverse library (CDL) of 7,800. Building on the results of this primary screening, we present here our medicinal chemistry approach that incorporates drug pair selection for combination screening by AI-based chemoinformatic and target identification with the use of activity-based protein profiling (ABPP) and photo-affinity labelling (PAL) probes coupled to quantitative proteomics.

**Consequences of VPS13B deficiency beyond the Golgi**

Júlia Mestres-Truyol and Jens Luders

*Mechanisms of Disease Programme, Institute for Research in Biomedicine (IRB Barcelona), The Barcelona Institute of Science and Technology (BIST), Baldiri Reixac 10, 08028 Barcelona, Spain*

Mutations in the vacuolar protein sorting 13 homolog B (VPS13B) gene cause autosomal recessive Cohen syndrome, a rare disease characterized by a wide range of symptoms including developmental delay, intellectual disability, postnatal microcephaly, progressive retinal dystrophy, intermittent neutropenia, and truncal obesity. Some of the clinical features in Cohen syndrome resemble the features observed in a group of disorders known as ciliopathies, which are characterized by defective formation and/or function of cilia. However, the molecular and cellular mechanisms underlying Cohen syndrome remain elusive. The emerging model regarding the function of the VPS13 family of proteins suggests that they localize at membrane contact sites, where they bridge membranes and form a channel for bulk lipid transport between organelles. In the case of VPS13B, previous work has focused on its role at the Golgi and associated membrane compartments, where it is primarily localized. Here, we show that VPS13B deficiency results in alterations in cellular lipid composition and distribution, and impairs multiple organelles including primary cilia. This is accompanied by the activation of major stress-related signaling pathways that compromise cell proliferation and homeostasis, potentially contributing to the disease phenotype. Together, our results suggest that unveiling how VPS13B deficiency is linked to Cohen syndrome clinical features, with the ultimate goal of identifying therapeutic opportunities, requires analyzing not only primary defects at the Golgi but also subsequent cellular responses in disease-relevant tissues.

**Bridging Genotype and Phenotype: The Role of *VPS13B* Missense Variants in Cohen Syndrome Pathogenesis**

J. Kühnisch^1^, G. Schottmann^2^, C. M. Almudéver^3^, W. Seifert^3^

*^1^Institute of Physiology, Brandenburg Medical School (MHB) Theodor Fontane, Brandenburg an der Havel, Germany*

*^2^Zentrum für Sozial-und Neuropädiatrie (DBZ), Vivantes Klinikum Neukölln, Berlin, Germany*.

*^3^Institute of Cell Biology and Neurobiology, Charité – Universitätsmedizin Berlin, corporate member of Freie Universität Berlin und Humboldt-Universität zu Berlin, Berlin, Germany*

Cohen syndrome (CS) is a rare, early-onset neurodevelopmental disorder characterized by postnatal microcephaly, intellectual disability, and variable clinical features. Biallelic protein-truncating (loss-of-function) variants in the *VPS13B* gene are the established genetic disease-causing mechanisms of CS. In contrast, the interpretation of VPS13B missense variants remains challenging due to the lack of genetic and functional evidence.

Here, we present a systematic approach to bridge genotype and phenotype for VPS13B missense variants of unknown significance (VUS). By employing VPS13B site-directed mutagenesis and overexpression in HeLa cells, we quantified subcellular localization with confocal microscopy and image analysis. Our results demonstrate that 13 out of 27 tested VUS disrupt VPS13B Golgi localization. Defective VPS13B Golgi localization is consistent with the established loss-of-function mechanism for CS. Notably, five of these VUS cluster within the VPS13B VAB domain that mediates membrane tethering.

Functional characterization of VPS13B missense variants is of clinical relevance. Our recent reclassification of theVPS13B missense VUS, p.Arg237Pro resulted in a likely pathogenic evaluation in a CS family (PMID: 39723426, DOI: 10.3389/fnins.2024.1488133). Thus, functional analysis will improve informed genetic counselling and update recurrence risk estimation.

Our study demonstrates the power of integrating VPS13B molecular function with clinical data to improve genetic variant interpretation. In the future, combining functional assays with structural modelling will refine genotype-phenotype correlation, diagnosis, and counselling for families affected by Cohen syndrome.

**Human Anterior Neural Organoids as a Promising Model for Cohen Syndrome**

Woong Sun and Prasad Renuka

*Department of Anatomy, Korea University College of Medicine, Seoul, Republic of Korea*

Neural organoids display three-dimensional structures that resemble in vivo neural architectures. Previously, we developed a novel protocol composed of two-dimensional (2D) neural induction and subsequent reaggregation of the induced neuroepithelial (NE) cells for culturing spinal cord organoids, enabling size control and recapitulating neural tube morphogenesis. In this study, we evaluated whether a similar approach is applicable to induce neural organoids that exhibit characteristics similar to the anterior regions of the brain, namely human anterior neural organoids (hANOs). By inducing anterior NE cells through dual-SMAD inhibition in 2D and reaggregating them, we achieved tube-forming morphogenesis similar to that of posterior spinal cord organoids. The transcriptome profiles of these hANOs most closely resembled those of the frontal cortex of human embryos at 20 weeks post-conception (PCW). Using this hANOs protocol, we investigated microcephaly phenotypes associated with Cohen syndrome (CS), which is caused by biallelic loss-of-function variants in the VPS13B gene. Deleting VPS13B in human pluripotent stem cells (hPSCs) resulted in Golgi dispersion in hPSCs and a slow onset of growth retardation in mutant hANOs, akin to CS patients with postnatal microcephaly. This delay is partly linked to reduced neuronal growth. Additionally, mature CS organoids exhibited enhanced hyper-excitability associated with an excitatory/inhibitory imbalance. In conclusion, this protocol is suitable for studying microcephaly phenotypes resulting from human genetic mutations due to its simplicity and scalability.

**Characterization of Mouse Models of Cohen Syndrome to Better Understand Disease Pathogenesis**

Binnaz Yalcin

*Institut National de la Santé et de la Recherche Médicale (INSERM), Inserm UMR1231, Université Bourgogne-Europe, 21000 Dijon, France*

In this study, we investigated how loss of the vacuolar protein-sorting gene **Vps13b**, whose mutations cause human Cohen syndrome, affects brain development and behavior. Using a constitutive *Vps13b* knockout mouse, we found that about half of homozygous pups die in the first postnatal week and that survivors display core features of the human disorder: postnatal microcephaly, altered memory, hypotonia, growth delay, and increased sociability. High-resolution 2D and 3D brain analyses revealed non-progressive structural anomalies: the hippocampus showed the most pronounced size reduction, while the motor cortex was specifically thinner in layer VI, whereas the fornix, fasciculus retroflexus, and cingulate cortex were unaffected. These neuroanatomical anomalies appeared after a window of neuronal loss during the first week of life and remained stable thereafter, with no further cell death. ***Vps13b*** is most highly expressed in the hippocampus and cortex, with a postnatal peak. Overall, the findings identify VPS13B as critical for neuronal survival, while the precise cause of cell loss remains to be determined in future studies.

### The 2025 Glenn Irvine Prize lecture

**Deciphering VPS13D’s function in physiology and disease**

Lucia Iannotta^1,2^, Martina Zambito^1,2^, Francesca Sala^1,2^, Bianca Esch^3^, Raffaella Morini^2^, Michela Matteoli^2,4^, Florian Fröhlich^3^, Paolo Colombi^5^ and Marianna Leonzino^1^^,2^

*^1^Institute of Neuroscience of Italian National Research Council (IN-CNR), c/o Humanitas Mirasole Spa, Rozzano (MI)*

*^2^IRCCS Humanitas Research Hospital, via Manzoni 56, Rozzano 20089, MI, Italy*

*^3^Bioanalytical Chemistry Section, Department of Biology/Chemistry, Osnabrück University, 49076 Osnabrück, Germany; Center of Cellular Nanoanalytics Osnabrück (CellNanOs), 49076 Osnabrück, Germany*

*^4^Department of Biomedical Sciences, Humanitas University, Via Rita Levi Montalcini 4, 20072 Pieve Emanuele, Milan, Italy*

*^5^Institute of Molecular Genetics Luigi Luca Cavalli-Sforza of Italian National Research Council (IGM-CNR), Pavia*

Compound heterozygous mutations on the VPS13D gene cause a devastating movement disorder characterized by heterogeneous symptoms including spastic ataxia, spastic paraplegia and dystonia. No cure is available for this disease, mainly because its pathogenetic mechanism is still largely obscure.

VPS13D is the only essential member of the VPS13 family, comprising evolutionary conserved bridge-like lipid transfer proteins (BLTPs) thought to mediate the expansion of cellular membranes by localizing at membrane contact sites and fueling lipids from a donor to an acceptor organelle [1].

Studies in flies and in human cells suggested a role for VPS13D in mitophagy, however the molecular mechanism underlying such function remains to be elucidated [2,3]. We are investigating the function(s) played by VPS13D using two synergistic approaches: 1) we developed a cell line bearing a degron tag fused to the endogenous VPS13D sequence and using this tool to uncover alterations in organellar physiology directly linked to VPS13D absence; 2) we generated human iPSC lines bearing some of the VPS13D mutations described in patients and we are exploiting neurons derived from these iPSCs to investigate how VPS13D malfunctions translate into the pathogenetic cascade leading to the disease.

The combination of these approaches is offering us invaluable insights on the protein function, revealing unexpected molecular pathways contributing to disease development.

## Poster presentations

### VPS13A

**Integrated phosphoproteomic analysis identifies pre-synaptic subcellular localization of potential vps13a interactors in isolated basal ganglia from *vps13a^-/-^* mice**

Veronica Riccardi,^1^ Oksana Sorokina,^2^ Enrica Federti,^1^ Flora Cozzolino,^3^ Maria Monti,^3^ Andreas Hermann,^4,5,6^ Angela Siciliano,^1,7^ Seth L. Alper,^8^ Jacopo Ceolan,^1,7^ Adrian Danek,^9^ Ruth H. Walker,^10,11^ Richard Pozzetto Huot,^1^ Tolunay Kavaz,^1^ Emilia Turco,^12^ Paola Defilippi,^12^ Kevin Peikert,^4,5,6^ Lucia De Franceschi^1,7^

*^1^Department of Engineering for Innovative Medicine- DIMI, University of Verona, Verona, Italy*

*^2^School of Informatics, University of Edinburgh, UK*

*^3^Department of Chemical Sciences, Federico II University of Napoli and CEINGE Biotecnologie Avanzate Franco Salvatore, Napoli, Italy*

*^4^Translational Neurodegeneration Section “Albrecht Kossel”, Department of Neurology, University Medical Center Rostock, University of Rostock, Rostock, Germany*

*^5^Center for Transdisciplinary Neurosciences Rostock (CTNR), University Medical Center Rostock, Rostock, Germany*

*^6^United Neuroscience Campus Lund-Rostock (UNC), Rostock site, Rostock, Germany*

*^7^Azienda Ospedaliera Universitaria integrata Verona, Verona, Italy*

*^8^Department of Medicine and Division of Nephrology, Beth Israel Deaconess Medical Center, and Department of Medicine, Harvard Medical School, Boston, Massachusetts, USA*

*^9^Neurologische Klinik und Poliklinik, LMU Klinikum, LMU München, Munich, Germany*

*^10^Department of Neurology, James J. Peters Veterans Affairs Medical Center, Bronx, NY, USA*

*^11^Department of Neurology, Mount Sinai School of Medicine, New York City, NY, USA*

*^12^Molecular Imaging Center – Dept. of Molecular Biotechnology and Health Sciences University of Torino; Italy*

VPS13A disease (previously known as chorea-acanthocytosis), is an ultra-rare autosomal recessive neurodegenerative disorder caused by mutations of the *VPS13A* gene encoding VPS13A protein, known also as chorein. Although our understanding of VPS13A structure and function has progressed, much about its homeostatic roles remains to be investigated. In basal ganglia from *Vps13a^-/-^* mice, we previously showed that the absence of Vps13a results in perturbation of proteostasis with accumulation of neurotoxic proteins as well as of active Lyn, a kinase of the Src family. To understand the impact of altered intracellular signaling pathways in basal ganglia from mice genetically lacking Vps13a, we carried out a phosphoproteomic analysis of immunoprecipitated phospho-Tyrosine (Tyr) enriched samples from isolated basal ganglia of wild-type and *Vps13a^-/-^* mice. Using a label free proteomic quantification, we identified 22 proteins less abundant and 25 more abundant in the mutant in comparison with wild-type. We then used the Synaptic proteome database, which combines 58 published synaptic proteomic datasets [1] to identify the subcellar localization of potential VPS13A interactors and found that they are highly represented in both post- and pre-synaptic compartments. Using the extensive network analysis, we further explored the possible molecular complexes and pathways that can be affected by VPS13A deficiency and identified a few pathways that are significantly overrepresented with VPS13A interactors. Among them are vesicle-mediated transport, synaptic vesicle exocytosis, intermediate filament organization and G-protein coupled receptor signaling pathways. Of note, the pre-synaptic network represented the most promising compartment for further exploration. To validate the identified over-represented pathways with VPS13A interactors, we carried out immunoblot analysis of pre-synaptic and post-synaptic fractions from wild-type and *Vps13a^-/-^* mice. In pre-synaptic fractions from isolated basal ganglia of *Vps13a^-/-^* mice, we found accumulation of both neurexin, and Rab 3, a small G-protein, suggesting a perturbation of the dynamic in dense core vesicles (DCVs) fusion with possible impact on neuromodulators release. Of note, neurexin has been previously shown to stabilize pre-synaptic assemblies after membrane protein-phospholipid membrane fusion. Indeed, we found an accumulation of synaptotagmin, which plays a key role in vesicle fusion with pre-synaptic membrane. Additionally, we found accumulation of synaptotagmin in the post-synaptic fraction from *Vps13a^-/-^* mice when compared to wild-type, suggesting a possible effect on post-synaptic vesicle trafficking. In *Vps13a^-/-^* mice, the perturbation of pre-synaptic vesicle dynamics is further corroborated by the upregulation of both heat shock protein (HSP) 70 and 90 in the pre-synaptic fraction from *Vps13a^-/-^* mice when compared to either post-synaptic fraction or wild-type fractions. This is of interest since previous studies have shown the important role of HSP70/90 in maintaining folded proteins and in protecting against the accumulation of neurotoxic proteins. Collectively our data highlight a possible role of VPS13A in pre-synaptic vesicles dynamic and in vesicle trafficking to the active zone. Further studies are planned to better characterize the VPS13A interactome in isolated basal ganglia from *Vps13a^-/-^* mice.

**Lack of the lipid-transfer protein VPS13A alters mouse motor function and impacts on synaptic proteomic profile: key factors of chorea-acanthocytosis pathogenesis**

A. Ramon-Lainez^1,2,3^, R. Jalal-Gharnati^1,2,3^, D. Ruiz-Camacho^1,2,3^, G. Besa-Selva^1,2,3^, M. Kucukerden^1,2,3^, J. Fernández-Irigoyen^4^, E. Santamaría^4^, J. Alberch^1,2,3^, M. Masana^1,2,3^, MJ. Rodríguez^1,2,3^

*^1^Department Biomedical Sciences, Institute of Neurosciences, School of Medicine and Health Sciences, University of Barcelona, Spain*

*^2^August Pi Sunyer Biomedical Research Institute (IDIBAPS), Barcelona, Spain*

*^3^Networked Center for Biomedical Research in Neurodegenerative Diseases (CIBERNED), Madrid, Spain*

*^4^Proteored-ISCIII, Proteomics Platform, Clinical Neuroproteomics Unit, Navarrabiomed, Departamento de Salud, UPNA, IdiSNA, Pamplona, Spain*

VPS13A disease (chorea-acanthocytosis; ChAc) is caused by loss-of-function mutations in the gene encoding the lipid-transfer protein VPS13A. In this disorder, the main neuropathological feature is the selective degeneration of the caudate nucleus and putamen linked to adult-onset chorea and dystonia. However, the function of VPS13A specifically in the basal ganglia has been poorly addressed. In this study, we evaluated the role of VPS13A in motor function and the proteomic profile of the cortico-striatal circuitry using a new complete VPS13A knock-out (KO) mouse model. We assessed cortico-striatal-dependent motor function of VPS13A-KO mice at 33 weeks of age by performing open field (OF), vertical pole (VP) and accelerating rotarod (ARR) tests. VPS13A-KO mice showed a decreased distance traveled in the OF and spent more time on the VP compared to WT littermates. In the ARR, VPS13A-KO mice exhibited reduced motor learning, with different affectation in male and female mice. Proteomic changes induced by VPS13A-KO were analyzed in striatum and cortex of 33-week-old mice. We identified 289 proteins deregulated by VPS13A-KO in the motor cortex and 116 in the striatum, with only a few proteins common to both brain regions. However, in both brain regions, functional bioinformatics analysis revealed significant alterations in processes such as energy metabolism, protein synthesis, and endocytic trafficking. Specifically, we found a VPS13A-KO-induced downregulation of proteins involved in mitochondrial function; while, at the synaptic level, VPS13A-KO induced overexpression of proteins involved in neurotransmission and synaptic function, along with alterations in cytoskeletal organization and local translation within neurites. Our findings demonstrate that the absence of VPS13A impacts on motor learning and coordination and alters the synaptic proteomic profile. Taken together, our results highlight the involvement of intracellular lipid-distribution mechanisms in cortico-striatal functional motor networks and synaptic protein content, which further contribute to understanding the specific vulnerability of basal ganglia in motor disorders such as ChAc.

Supported by the Ministerio de Ciencia e Innovación (grant number: PID2023-150728OB-I00 to JA and MJR, PID2021-124896OA-I00 and CNS2023-14399 to MM) and Fundación ChAc (Spain). The GlioLight project has received funding from the European Innovation Council under grant Agreement 101129705 (MM).

**Neuronal loss of VPS13A lipid transfer protein disrupts DAG/PKC signaling in a chorea-acanthocytosis model**

G. Besa-Selva^1,2,3^, E. García-García^1,2,3^, A. Ramón-Lainez^1,2,3^, G. Escaramís^1,4^, P. Garcia-Segura^1,2,3^, C. Malagelada^1,2,3^, E. Martí^1,2^, M. Masana^1,2,3^, J. Alberch^1,2,3^, M. J. Rodríguez^1,2,3^.

*^1^Department Biomedical Sciences, Institute of Neurosciences, School of Medicine and Health Sciences, University of Barcelona, Spain*

*^2^August Pi Sunyer Biomedical Research Institute (IDIBAPS), Barcelona, Spain*

*^3^Network Center for Biomedical Research in Neurodegenerative Diseases (CIBERNED), Madrid, Spain*

*^4^Biomedical Research Networking Center for Epidemiology and Public Health (CIBERESP), Spanish Ministry of Science and Innovation, Madrid, Spain*

Chorea acanthocytosis (ChAc) is an ultrarare inherited neurodegenerative disease caused by a VPS13A gene mutation, resulting in the loss of the VPS13A protein. This leads to a selective and progressive neurodegeneration of the striatum and, consequently, to a dysfunction of the corticostriatal pathway. VPS13A functions as a bulk lipid transporter between different organelles, suggesting that neuronal lipid dyshomeostasis may be a key pathological process in ChAc. However, the precise role of VPS13A in lipid function in neurons remains unknown.

To elucidate this, we established a shRNA-based knockdown (KD) mouse model and investigated the effect of VPS13A KD on mouse brain lipid concentration in an open-hypothesis lipidomic analysis. We identified 173 different glycerophospholipid and sphingophospholipid species, along with some of their precursors and derivatives, in the brain tissue of KD and Control (Ctrl) mice. Analysis of differential concentration revealed significantly elevated levels of 14 different diacylglycerol (DAG) species in KD brain tissue. Then, we explored the metabolic pathways and downstream molecular pathways that are related to the altered diacylglycerol levels and found that VPS13A knockdown induces a decrease in protein kinase C (PKC)β concentration but an increase in PKCα/β phosphorylation in cultured neurons. Specific pharmacological inhibition of PKCβ induced aberrant neuronal morphology in VPS13A KD neurons but not in controls. These results provide evidence of the important role lipid cellular distribution mechanisms play in regulating synaptic plasticity processes. Our results also uncover the deregulation of diacylglycerol/PKC signaling as a new pathophysiological mechanism driving neuronal dysfunction and degeneration in ChAc.

Supported by the Ministerio de Ciencia e Innovación y la Agencia Estatal de Investigación (grant number: PID2023-150728OB-I00 to JA and MJR, PID2021-124896OA-I00 and CNS2023-143999 to MM) and Fundación ChAc (Spain). The GlioLight project has received funding from the European Innovation Council under grant Agreement 101129705 (M.M.).

**Neuronal deficiency of the lipid-transfer protein VPS13A impairs mitochondrial homeostasis and function**

A. Ramon-Lainez^1,2,3^, E. García-García^1,2,3^, G. Besa-Selva^1,2,3^, D. del Toro^1,2,3^, L. Valls^2^, G. Garrabou^2^, S. Ginés^1,2,3^, M. Masana^1,2,3^, J. Alberch^1,2,3^, M. J. Rodríguez^1,2,3^

*^1^Department Biomedical Sciences, Institute of Neurosciences, School of Medicine and Health Sciences, University of Barcelona, Barcelona, Spain*

*^2^August Pi Sunyer Biomedical Research Institute (IDIBAPS), Barcelona, Spain*

*^3^Network Center for Biomedical Research in Neurodegenerative Diseases (CIBERNED), Madrid, Spain*

VPS13A is an intermembrane lipid-transfer protein whose loss-of-function mutations, causing the protein depletion, lead to chorea-acanthocytosis (ChAc), an ultra-rare autosomal recessive neurodegenerative disorder. The main neuropathological characteristic of ChAc is the selective degeneration of the striatum linked to the progressive adult-onset chorea and dystonia. Yet, the underlying pathological mechanisms are poorly understood. Our group recently described that VPS13A colocalizes with endoplasmic reticulum and mitochondria organelle in neurons. Besides, mitochondrial fragmentation has been described in VPS13A knockout cell line models. Here, we aimed to investigate the impact of neuronal VPS13A in mitochondrial homeostasis and function. To identify VPS13A-interacting partners in the mouse brain, we performed protein immunoprecipitation followed by mass spectrometry analysis. We used a shRNA-based VPS13A knockdown (KD) model in primary neuronal cultures to assess the effects of VPS13A KD in mitochondrial morphology, dynamics, and function. Mitochondrial metabolism was evaluated using Seahorse Analytics. Potential dysfunction mechanisms and mitochondrial stress were analyzed measuring mitochondrial membrane potential and reactive oxygen species, respectively. Our mass spectrometry analysis revealed that VPS13A mainly interacts with proteins involved in mitochondrial metabolism. In neuronal cultures, VPS13A downregulation reduced mitochondrial size and decreased mitochondrial fusion and fission dynamics. Energy metabolism studies showed decreased aerobic and anaerobic metabolism, reduced ATP production, impaired metabolic potential and altered general metabolic profile in VPS13A KD neurons. Furthermore, VPS13A KD induced mitochondrial membrane hyperpolarization and reduced the mitochondrial membrane potential depolarization speed. Moreover, we observed a higher production of mitochondrial reactive oxygen species in VPS13A KD neurons, indicative of increased oxidative stress. Taken together, our findings provide evidence that reduced VPS13A levels in neurons alter mitochondria morphology, disrupt energy metabolism, impair mitochondrial function, and elevate mitochondrial stress. These results highlight the key role of cellular lipid distribution in maintaining mitochondrial homeostasis and function. They also contribute to unveil the pathophysiological mechanisms underlying ChAc.

Supported by the Ministerio de Ciencia e Innovación la Agencia Estatal de Investigación (grant number: PID2023-150728OB-I00 to JA and MJR, PID2021-123732OB-I00 to SG, PID2021-124896OA-I00 and CNS2023-14399 to MM) and Fundación ChAc (Spain). The GlioLight project has received funding from the European Innovation Council under grant Agreement 101129705 (M.M.).

**VPS13 proteins are essential to maintain ER membrane lipid composition and overall cellular homeostasis**

Paula Sànchez-Fernàndez-de-Landa^1,2,3^, Marc Beltrà^1,2,3^, Katerina Danezi^1,2,3^, Antonio Zorzano^1,2,3^

*^1^Institute for Research in Biomedicine (IRB Barcelona), The Barcelona Institute of Science and Technology, Barcelona, Spain*

*^2^Department of Biochemistry and Molecular Biomedicine, Faculty of Biology, University of Barcelona, Spain*

*^3^CIBER de Diabetes y Enfermedades Metabólicas Asociadas (CIBERDEM), Instituto de Salud Carlos III, Barcelona, Spain*

Membrane contact sites (MCS) are important hubs for biological processes where specific proteins are recruited to ensure cellular functionality. VPS13 proteins are lipid transporters that localize at MCS, functioning as membrane bridges and allowing lipids to slide within their groove between two close bilayers. In addition, pathogenic variants from VPS13 mammalian homologs are linked to lipid transport impairment causing neurodegenerative disorders. VPS13A and VPS13D interact with the endoplasmic reticulum (ER) through their N-terminal domain, connecting it to multiple MCS. Current models propose the ER as the source of lipids from where VPS13A and VPS13D supply adjacent organelles. Nevertheless, conclusive evidence on how VPS13 proteins contribute to ER homeostasis is still missing. The main objective of our study is to understand if VPS13A and VPS13D loss of function could lead to ER dysfunction, disrupting overall cellular homeostasis and promoting pathology.

Lipidomic analysis shows that either VPS13A or VPS13D deficient fibroblasts present an accumulation of specific lipid families in ER-enriched fractions, together with enhanced unfolded protein response (UPR) and increased ER branching via IRE1 signaling, implying ER lipid bilayer stress. Consistently, specific lipid manipulations rescue the UPR activation and mitigates the altered ER branching in both VPS13A and VPS13D knockdown cell lines, demonstrating a correlation between ER membrane lipid composition and its homeostasis. Furthermore, upon these VPS13 proteins repressions, mitochondria become fragmented and cells increase their susceptibility to apoptosis. Altogether, these results identify VPS13 proteins as important regulators of ER lipid remodeling and overall cellular functionality.

### VPS13B

**A Novel Cohen Syndrome Zebrafish Model Reveals a Possible Relationship Between Vps13b and Cilia**

Alessandra Guerrero Samanidis^1^, Max Duong Phu^1^, Júlia Mestres^2^, Marta Lovera^2^, Martin Burkhalter^1^, Dunja Lukovic^3^, Jens Lüders^2^, Melanie Philipp^1^

*^1^Department of Experimental and Clinical Pharmacology and Pharmacogenomics, Division of Pharmacogenomics, University Hospital Tübingen, Tübingen, Germany*

*^2^Mechanisms of Disease Program, Institute for Research in Biomedicine (IRB Barcelona), The Barcelona Institute of Science and Technology (BIST), Barcelona, Spain*

*^3^Retinal degeneration Lab, Department of Animal Production and Health, Public Veterinary Health and Food Science and Technology School of Veterinary Medicine, Universidad Cardenal Herrera-CEU, CEU Universities, Alfara del Patriarca, Valencia, Spain*

The rare autosomal recessive disease Cohen Syndrome (CS) is characterized by ocular defects, microcephaly, neutropenia, learning disabilities, muscular hypotonia, and truncal obesity. Pathogenetically, CS is caused by mutations in the Vacuolar Protein Sorting Protein 13 B (VPS13B). Recent publications suggest that VPS13B facilitates lipid transport between compartments of the Golgi apparatus or at Golgi-lipid droplet membrane contact sites. It has furthermore, been linked to ER-Golgi transport. The exact functions of the protein and the underlying pathomechanisms of the disease, however, remain to be fully elucidated. There are several animal models mimicking selected clinical symptoms of the disease. However, most of them do not resemble the full complexity of CS pathology.

Our group established a novel zebrafish Vps13b loss-of-function model imitating many of the clinical manifestations observed in CS patients. Live phenotype analyses together with Sudan black B staining showed that the three critical clinical symptoms of CS, microcephaly, ocular defects, and neutropenia, are present in Vps13b-depleted zebrafish embryos. Moreover, immunofluorescence experiments revealed fewer motoneurons as well as fewer muscle fibers in Vps13b-deficient embryos consistent with impaired motoric function in CS patients. Closer analysis of the developing eye revealed coloboma, severe retinal lamination defects and a failure to differentiate into committed retinal cell types. This was accompanied by reduced proliferation and simultaneously increased apoptosis in the eye. Biochemical analyses demonstrated impaired lipid handling and transport in Vps13b-depleted embryos. Interestingly, we found Vps13b to be expressed in various ciliated tissues. Detailed analysis revealed cilia defects in certain organs and dysregulation of genes associated with ciliary lipid-modified protein transport.

In summary, we present a novel zebrafish CS model resembling most of the patients’ clinical manifestations, which we could link to defects in cilium formation.

**Neuromuscular phenotyping in neonatal and adult mouse models of Cohen syndrome indicates a potential central origin of hypotonia**

Xiang Wu^1,2^, Charlotte Montillot^1^, Emilia Skunotua^1^, Hong Li^2^, Stephan Collins^1^ and Binnaz Yalcin^1^

*^1^Institut National de la Santé et de la Recherche Médicale (INSERM), Inserm CTM UMR1231, Université Bourgogne-Europe, 21000 Dijon, France*

*^2^Ningbo Medical Center Li Huili Hospital, Health Science Center, Ningbo University, Ningbo, 315000, PR China*

The vacuolar protein sorting-associated protein 13B (VPS13B) is a large, highly conserved protein. Disruption of VPS13B causes autosomal recessive Cohen syndrome, a rare disorder characterized by microcephaly and intellectual disability, along with other features including hypotonia. However, the mechanisms by which VPS13B disruption leads to muscle dysfunction remain completely unexplored.

To gain insights into the myopathogenesis of Cohen syndrome, we systematically reviewed the literature and identified 47 reports of VPS13B-related cases. Among these, hypotonia was reported in 100% of male cases (17 papers) and in 75% of female cases; the latter figure should be interpreted cautiously due to the small sample size (only four reports).

To model the recessive VPS13B defect, we used a mouse model generated by homologous recombination to assess muscle function in both adult and neonatal animals. Adult mutant homozygous males showed muscular weakness (–14%, P = 0.002) in both forelimbs and hindlimbs on the grip test. This was confirmed by the bar holding test, where the latency to fall was 2.5-fold shorter (P = 0.00001) compared to matched wild-type mice. Adult mutant homozygous females were also hypotonic. They showed reduced grip strength compared to wild-type females for forelimbs (–20%, P = 0.007) and for both forelimbs and hindlimbs (–14%, P = 0.006). This was confirmed in the bar holding test, where the latency to fall was four-fold shorter (P = 0.028) compared to matched wild-type females.

To assess neonatal muscle function from postnatal day 2 (P2) to P21, we developed a new pipeline for neuromuscular phenotyping comprising 16 distinct assays. These evaluate motor skills (coordination, strength, and endurance), developmental milestones (ear and eye opening, tooth eruption, fur appearance), neuromuscular reflexes (eyelid reflex, righting reflex, forelimb and hindlimb grasping reflex, negative geotaxis, cliff avoidance) and behavior such as the open-field arena paradigm. Initial observations at P21 indicate that mutant homozygous mice show reduced motor strength and coordination in the bar holding and negative geotaxis assays. This suggests that muscle anomalies start early in mice recapitulating the human manifestations. Work is ongoing to determine the time window in which these anomalies first appear within the P2-P21 range.

Next, we assessed neuroanatomical changes and found specific morphological alterations in the brain emerging after birth. Notably, the motor cortex was thinner in layer VI and was accompanied by increased neuronal death. These findings point to defective thalamocortical modulation, potentially weakening muscle tone. Ongoing work aims to validate this mechanism using in vivo electrophysiology or calcium imaging of layer VI neurons during muscle function assays. Alternatively, electromyography will be used to detect reduced baseline muscle activity despite intact contractility.

In conclusion, these preliminary findings provide new insights into the muscle biology of VPS13B and highlight a likely central contribution to the hypotonia observed in Cohen syndrome, which remains to be validated.

### XK disease

**XK disease (McLeod syndrome): an autopsy series of six cases**

Klaudia F. Laborc^1,2,5,7,8^, Claudia De Sanctis^1,2,5,7,8^, Emma L. Thorn^1,2,5,7,8^, Lily Y. C. Chiu^1,2,5,7,8^, Victoria Flores Almazan^1,2,5,7,8^, Quazi I. Hossain^1,2,5,7,8^, Stephanie McQuillan^1,2,5,7,8^, Kourtni Lind-Watson^1,2,5,7,8^, Yolfrankcis Mendez^2,3^, Anya C. McGoldrick^2,3^, Jamie M. Walker^1,2,5,9^, Melissa J. Nirenberg^3,4^, Ruth H. Walker^3,4^, John F. Crary^1,2,5,6,7,8^

*^1^Department of Pathology, Molecular and Cell-Based Medicine, Icahn School of Medicine at Mount Sinai, New York, USA*

*^2^Neuropathology Brain Bank & Research CoRE, Icahn School of Medicine at Mount Sinai, New York, USA*

*^3^James J. Peters Department of Veterans Affairs Medical Center, Bronx, USA*

*^4^Department of Neurology, Icahn School of Medicine at Mount Sinai, New York, USA*

*^5^Nash Family Department of Neuroscience, Icahn School of Medicine at Mount Sinai, New York, USA*

*^6^Department of Artificial Intelligence & Human Health, Icahn School of Medicine at Mount Sinai, New York, USA*

*^7^Ronald M. Loeb Center for Alzheimer’s Disease, Icahn School of Medicine at Mount Sinai, New York, USA*

*^8^Friedman Brain Institute, Icahn School of Medicine at Mount Sinai, New York, USA*

*^9^Glenn Biggs Institute for Alzheimer’s & Neurodegenerative Diseases, University of Texas Health Science Center at San Antonio, San Antonio, USA*

Introduction: XK disease, formerly known as McLeod syndrome, is one of the core neuroacanthocytosis syndromes. These very rare neurodegenerative diseases primarily affect the caudate nucleus, and are often accompanied by red blood cell changes (acanthocytosis). Patients can present with a spectrum of symptoms, including chorea, parkinsonism, tics, peripheral neuropathy and myopathy, cognitive and psychiatric symptoms, and seizures. The pathophysiology of this disease is not understood, and very few neuropathological cases have been reported. Our aim is to systematically examine the neuropathological features of XK disease using a wide range of neuropathological markers.

Methods: We performed detailed gross and histological examination on post-mortem formalin-fixed tissue from five genetically confirmed XK disease patients and six age- and sex-matched controls. All cases underwent an extensive neuropathological workup. Microscopic analysis focused on dorsolateral frontal cortex, basal ganglia, and midbrain. A battery of neurohistological stains were completed including hematoxylin-eosin, β-amyloid, phosphorylated tau, Bielschowsky silver, α-synuclein, TDP-43, p62, ubiquitin, and CD-68.

Results: Macroscopic examination revealed variable mild-moderate external cortical atrophy. On coronal sections, moderate dilation of the ventricles and severe atrophy of the anteromedial striatal structures was found. Microscopic findings showed significant atrophy of the caudate nucleus with vacuolized neuropil, variable neuronal loss and marked astrogliosis. Additionally, the cases presented variable degrees of Alzheimer’s disease neuropathologic changes.

Conclusion: Our study is the largest histopathological case series of XK disease to date. We anticipate that, in addition to our current findings, our planned lipidomic and transcriptomic studies will reveal molecular pathways involved in the pathophysiology of this very rare disorder.

Funding: NA Advocacy, USA.

**Brain Sphingolipid and Phospholipid Levels Are Altered in XK disease**

Gabriel Miltenberger-Miltenyi^1,2^, Vasco A. Conceição^1-2^, Klaudia F. Laborc^3-7^, Claudia De Sanctis^3-7^, Emma Thorn^3-7^, Lily Yu-Chia Chiu^3-7^, Melissa J. Nirenberg^4,5,8,9^, John F. Crary^3-7^, Ruth H. Walker^4,5,8,9^

*^1^Laboratório de Genética, Faculdade de Medicina, Universidade de Lisboa, Lisbon, Portugal*

*^2^Reference Center on Lysosomal Storage Diseases, Hospital Senhora da Oliveira, Guimarães, Portugal*

*^3^Department of Pathology, Molecular, and Cell Based Medicine, Icahn School of Medicine at Mount Sinai, New York, New York, USA*.

*^4^Neuropathology Brain Bank & Research CoRE, Icahn School of Medicine at Mount Sinai, New York, USA*

*^5^Friedman Brain Institute, Icahn School of Medicine at Mount Sinai, New York, NY, USA*

*^6^Ronald M. Loeb Center for Alzheimer’s Disease, Icahn School of Medicine at Mount Sinai, New York, USA*

*^7^Department of Artificial Intelligence & Human Health, Icahn School of Medicine at Mount Sinai, New York, USA*

*^8^Department of Neurology, James J. Peters Veterans Affairs Medical Center, Bronx, NY, USA*

*^9^Department of Neurology, Icahn School of Medicine at Mount Sinai, New York, NY, USA*

Background: XK-disease (McLeod syndrome) is an X-linked multisystemic neurodegenerative disorder caused by mutations in the XK gene that codes for the lipid scramblase XK.

Objectives: To describe the lipidomic spectrum in postmortem brain tissue from XK patients.

Methods: We measured levels of 593 lipid species by HPLC-MS in the caudate nucleus (CN), putamen, and dorsolateral prefrontal cortex (DLPFC) from postmortem tissues of five XK and six control patients. We compared levels between groups using general linear mixed-effects models and performed multiple-comparison correction using FDR.

Results: We observed increased levels of ceramide, monoacyl- and triacylglycerol, phosphatidylserine in the CN of XK patients. Acylated phosphatidylglycerol levels were reduced in both CN and putamen. Acyl carnitine, dihydrosphingomyeline and monosialodihexosylganglioside were reduced while N-acyl phosphatidylethanolamine was increased in the DLPFC. N-acyl serine was reduced in all three regions in XK patients.

Conclusions: Our findings provide initial evidence of abnormal sphingolipid and phospholipid concentrations in the brains of patients with XK. Our findings support the role of XK of impaired lipid metabolism and in apoptosis as reported in recent studies on cellular and animal models.

Funding: NA Advocacy, USA.

### BLTP2

**In-silico Investigation of bulk lipid transport mediated by BLTP2**

Yara Ahmed^1^, Hailey Eng^2,3^, Elizabeth Conibear^2,3,4^, Stefano Vanni^5^

*^1^Department of Biology, University of Fribourg, Switzerland*

*^2^Centre for Molecular Medicine and Therapeutics, British Columbia Children’s Hospital Research Institute, University of British Columbia, Vancouver, Canada*

*^3^Department of Cellular and Physiological Sciences, University of British Columbia, Vancouver, Canada*

*^4^Department of Medical Genetics, University of British Columbia, Vancouver, Canada*

*^5^NCCR Bioinspired Materials, University of Fribourg, Fribourg, Switzerland*

BLTP2, a bridge-like lipid transport protein, is highly conserved among eukaryotes. It is composed of repeating β-groove (RBG) domains that assemble into a rod-like structure harboring a continuous hydrophobic channel along its length. BLTP2 predominantly localizes to endoplasmic reticulum–plasma membrane (ER–PM) contact sites. This structure and localization equip BLTP2 to mediate bulk lipid transfer across the cytosol, nevertheless, the underlying molecular mechanism remains unknown. We resort to molecular dynamics (MD) simulations at various atomic resolutions to explore the process of BLTP2-mediated lipid transport. We show preliminary results on the binding interfaces of BLTP2 with the membranes. Moreover, we observe spontaneous lipid desorption at the N-terminal interface of BLTP2 at the ER membrane. Additionally, we investigate potential interactors and/or adaptors that could stabilize the C-terminal interaction with the PM or facilitate lipid desorption in this region.

## Meeting summary

The recognition of the new BLPT protein family, and of XK as having functional and structural relationship, at least to VPS13A, has resulted in significant progress in the field. This has been greatly facilitated by novel modeling methodologies. The various members of the BLTP family share structural and functional features in lipid transport between specific intracellular organelles; recent work has provided insights into regulation of lipid transport based upon structural features which is anticipated to lead to understanding of dysfunction in disease states. There is significant variation in clinical disorders related to pathogenic variants of this gene family, which can result variously in neurodevelopmental (*VPS13B*, *BLTP1)* or neurodegenerative disorders affecting the caudate nucleus/putamen (*VPS13A, XK)*, substantia nigra pars compacta (*VPS13C*), or cerebellum (*VPS13D)*. In some disorders (*VPS13D*), there is clear phenotype-genotype correlation, while this is not the case in others, which also should ultimately reveal specifics of protein functioning. VPS13D appears to be the only essential member of the family, while there appears to be some redundancy in the functions of VPS13A and VPS13C. The identification of interacting protein partners, and the downstream consequences of protein absence or dysfunction upon organelles such as mitochondria, may well provide information on the cellular processes which are affected, resulting in neurodevelopmental or neurodegenerative issues, such as abnormal autophagy. Data from model systems, including organoids, zebrafish, and mouse models, demonstrate the effects of protein absence or dysfunction at a higher level. These can ultimately be validated by comparison with studies in humans, for example from peripheral blood samples and from postmortem tissue. Examination of human blood continues to be a focus, particularly in VPS13A and XK diseases, with the goal of identifying biomarkers to facilitate diagnosis and to track disease progression, and also to improve understanding of membrane dysfunction in the generation of acanthocytes. These studies will be critical for clinical trial readiness, and to examine the efficacy of any potential therapeutic agents.

## The virtual VPS13 Forum sessions

Following this in-person symposium, we have continued the online *VPS13 Forums*. These virtual meetings fill the gap between the biannual in-person symposia and are dedicated to all aspects of research, ranging from bench to bedside. Table 1 summarizes topics and speakers of the *VPS13 Forums* 12–22 (for Forums 1–11, see [1]). In addition to giving access to real-time presentations and discussions for those who were unable to attend the in-person symposia, speakers may share very recent results prior to publication, thus enhancing collaborative efforts and the opportunity to move the research field forward more expeditiously. Affected patients, carers, and family members also attend these meetings. The discussions which originate from these sessions facilitate prioritization of research goals, and generate clinical insights which otherwise may be missed, especially given the rarity of these disorders [2].

**Table 1:** ***VPS13 Forum* Virtual Meetings 11–22 from March, 2023 to present**. For invitations to future *VPS13 Forum* sessions, please contact kevin.peikert@med.uni-rostock.de.


TOPIC	SPEAKERS

Muscle and Nerve	March 27, 2023	Anne Buchberger (Department of Neurology, Klinikum rechts der Isar, Technical University Munich, Munich, Germany) Hans Jung (Department of Neurology, University Hospital and University Zurich, Zurich, Switzerland) Marina Melone^1^ & Pietro Chiurazzi^2^ (^1^Center for Rare Neurological Diseases, HCP representative for MetabERN and EURO-NMD networks; Department of Advanced Medical and Surgical Sciences, InterUniversity Center for Research in Neurosciences, University of Campania «Luigi Vanvitelli», Naples Italy; ^2^Sezione di Medicina Genomica – Genomic Medicine Dipartimento di Scienze della Vita e Sanità Pubblica Università Cattolica del Sacro Cuore Roma, Italy) Lucia De Franceschi (Department of Medicine, University of Verona & AOUI Verona, Policlinico GB Rossi, Verona, Italy)

Bridge-like lipid transfer proteins	May 22, 2023	Ruth H. Walker (Department of Neurology, Mount Sinai School of Medicine, New York, and James J Peters Veterans Affairs Medical Center, New York, NY, USA) Arash Bashirullah (Division of Pharmaceutical Sciences, University of Wisconsin-Madison, WI, USA) Martin Graef (Department for Molecular Biology and Genetics, Cornell University, Ithaca, NY, USA)

Linking the Patient and Medical/Science Communities	July 31, 2023	Medical Q&A and Discussion Panel: Adrian Danek, Ginger Irvine, Susan Wagner, Joy Willard-Williford, Bob Metzger, Ricky Ditzel, Ruth Walker

Homburg Conference Summary and Medical Q&A	November 20, 2023	Summary and follow-up discussion of the 11th International Meeting in Neuroacanthocytosis Syndromes Medical and Q&A for patients/families/caregivers

Research progress in VPS13B related Cohen Syndrome	January 22, 2024	Ashley Waterman (Cohen Syndrome Research Foundation, California, LA, USA) Heng Wang, (DDC Clinic for Special Needs Children, Middlefield, OH, USA) Fabrizio Vacca (Jules Gonin Eye Hospital, University of Lausanne, Lausanne, Switzerland) Woong Sun and Jungmin Choi (Department of Biomedical Sciences, Korea University, Seoul, Korea) Binnaz Yalcin (Centre for Translational and Molecular medicine, INSERM U1231, Dijon, France)

“Between Huntington’s and Parkinson’s?” Examining the links between Huntington’s and Parkinson’s diseases and neuroacanthocytosis syndromes (XK and VPS13A diseases)	April 29, 2024	Xueyi Li (Harvard Medical School, Charlestown MA, USA) Bernhard Landwehrmeyer (University Department of Neurology and Huntington-Zentrum, Ulm, Germany) Michael Schwake (Department of Chemistry, Biochemie III, University of Bielefeld, Bielefeld, Germany)

Nutritional challenges among people with feeding dystonia and Medical Q&A	July 29, 2024	Martin Paucar (Department of Clinical Neuroscience, Karolinska Institutet, and Department of Neurology, Karolinska University Hospital, Stockholm, Sweden) Charlotta Rubin (Department of Neurology, Karolinska University Hospital, Stockholm, Sweden) Josefin Kyhle (Department of Neurology, Karolinska University Hospital, Stockholm, Sweden) Elina Tripoliti (National Hospital for Neurology and Neurosurgery, London, UK)

VPS13C-associated Parkinsonism & VPS13C protein in lysosomal function	October 28, 2024	Lina Stauber (Department of Neurology, University of Regensburg, Germany) Leonie Schroeder (Department of Neurology, Feinberg School of Medicine, Northwestern University, Chicago, USA and Department of Chemistry/Biochemistry III, University of Bielefeld, Bielefeld, Germany) Xinbo Wang & Pietro De Camilli (Department of Neuroscience, Yale University School of Medicine, CT, USA)

Lipid Transfer and Calcium	February 10, 2025	André Nadler (Max Planck Institute of Molecular Cell Biology and Genetics, Dresden, Germany) Dajana Grossmann (Translational Neurodegeneration Section “Albrecht Kossel”, University of Rostock, Germany)

Medical Q&A	April 28, 2025	Medical Q&A and Discussion Panel: Julie Kerner, Alzbeta Mühlbäck, Elina Tripoli, Ruth H. Walker

Novel functions of VPS13 and XK proteins	July 28, 2025	Shiqi Liao & Goutam Kumar Tanti (Department of Neurology, Klinikum rechts der Isar, School of Medicine and Health, Technical University of Munich, Munich, Germany) Paula Sanchez (Institute for Research in Biomedicine (IRB Barcelona), The Barcelona Institute of Science and Technology, Baldiri Reixac, Barcelona, Spain) Karin Reinisch (Department of Cell Biology, Yale University School of Medicine, New Haven, CT, USA) Angelika Harbauer (Max Planck Institute for Biological Intelligence, Martinsried, Germany, and Technical University of Munich, Institute of Neuronal Cell Biology, Munich, Germany)


Involvement of the affected community enhances their engagement, their motivation to engage in clinically-oriented research (such as blood sample and brain donation), and gives them hope that researchers are working ultimately towards a cure for their diseases.
